# CHA_2_DS_2_-VASc Score as a Predictor of New-Onset Atrial Fibrillation After Catheter Ablation of Typical Atrial Flutter

**DOI:** 10.3389/fphys.2020.00558

**Published:** 2020-06-10

**Authors:** Fei Liu, Zechang Xin, Khalid Bin Waleed, Yajuan Lin, Gary Tse, Andrew Luhanga, Yuanjun Sun, Lianjun Gao, Xiaomeng Yin, Yunlong Xia

**Affiliations:** ^1^Department of Cardiology, The First Affiliated Hospital of Dalian Medical University, Dalian, China; ^2^Faculty of Medicine, Dalian Medical University, Dalian, China; ^3^Department of Cardiology, Fuwai Hospital Chinese Academy of Medical Sciences Shenzhen, Shenzhen, China

**Keywords:** atrial fibrillation, atrial flutter, catheter ablation, CHA_2_DS_2_-VASc score, risk factor

## Abstract

**Purpose:**

Cavotricuspid isthmus (CTI) ablation is an effective procedure for typical atrial flutter (AFL), but patients remain at an elevated risk for developing new atrial fibrillation (AF). Currently, there are limited data on the utility of CHA_2_DS_2_-VASc score to predict new-onset AF after typical AFL ablation. In this study, we assessed whether the CHA_2_DS_2_-VASc score is a useful predictor of new-onset AF after CTI ablation in typical AFL patients without a prior history of AF.

**Methods:**

This was a retrospective study of 103 typical AFL patients with no prior history of AF, who underwent successful CTI ablation. The endpoint was occurrence of new-onset AF during follow-up.

**Results:**

During a mean follow-up period of 24.6 ± 16.9 months, at least one episode of AF occurred in 33 (32%) patients. Multivariate Cox regression analysis revealed that CHA_2_DS_2_-VASc score (hazard ratio = 1.736; 95% confidence interval = 1.370–2.201; *P* < 0.001) was significantly associated with postablation new-onset AF (area under the curve = 0.797). A cutoff value of three stratified these patients into two groups with different incidences of postablation new-onset AF (67.9 vs. 18.7%, *P* < 0.001).

**Conclusion:**

The CHA_2_DS_2_-VASc score is a useful tool for the prediction of new-onset AF after ablation of typical AFL. Patients with CHA_2_DS_2_-VASc score ≥3 are more likely to develop new-onset AF and should be monitored more closely.

## Introduction

Cavotricuspid isthmus (CTI) ablation by radiofrequency is considered as the first-line therapy, and its reported success rate exceeds 90% for rhythm control in typical atrial flutter (AFL; Spector et al., 2009). However, the occurrence of new-onset atrial fibrillation (AF) is not uncommon after successful CTI ablation (Celikyurt et al., 2017). It is clinically important to predict subsequent new-onset AF to optimize the management strategies including surveillance, continuous anti-arrhythmic drug (AAD), anticoagulation, or prophylactic pulmonary vein isolation (PVI) during or after CTI ablation (Romanov et al., 2018).

The mechanisms responsible for the development of postablation new-onset AF are still unclear in AFL patients. Published data suggested that common comorbidities associated with electrophysiological triggers and substrate leading to cardiac electrical and structural remodeling might be responsible for arrhythmia incidence (Waldo, 2013). Chen et al. (2015) investigated HATCH score and purposed an association with an incident of new-onset AF after typical AFL ablation. However, CHA_2_DS_2_-VASc is a more commonly used and clinical scoring system than HATCH, and its use for predicting new-onset AF after AFL ablation has not been explored. Moreover, many studies indicated that most components of CHA_2_DS_2_-VASc score are associated with cardiac remodeling, and a higher score correlated with a greater degree of cardiac structural and electrical remodeling (Park et al., 2011; Kornej et al., 2014; Ribo et al., 2015; Wang et al., 2016). Here, we hypothesized that patients with a higher CHA_2_DS_2_-VASc score might be associated with postablation new-onset AF in typical AFL patients. Therefore, this study was carried out to assess the usefulness of the CHA_2_DS_2_-VASc score as a predictor of postablation new-onset AF in AFL patients without a prior history of AF.

## Materials and Methods

### Study Population

The population of this study consisted of 124 newly diagnosed AFL patients who underwent first-time successful CTI ablation at the First Affiliated Hospital of Dalian Medical University between March 2012 and December 2018. In the present study, a successful CTI ablation was defined by a bidirectional conduction block over the isthmus. All admitted patients in our hospital were carefully interviewed for present and past clinical history. ECG tracings including 12-lead ECG and available 24-h Holter were carefully reviewed before ablation procedure to determine prior history of AF. Patients with prior AF, valvular heart disease, or repeated ablations or those AFL patients with clinical history >1 month, had non-CTI-dependent circuits, were in AAD use after the ablation, or had incomplete follow-up history were excluded. Data on demography, comorbid conditions, echocardiographic parameters, CHA_2_DS_2_-VASc scores, and HATCH scores were obtained from electronic medical records before ablation in all patients. The First Affiliated Hospital of Dalian Medical University ethics review committee approved this study, and all patients provided written informed consent before enrollment.

### Definitions

Atrial flutter was defined as visible and regular inverted flutter waves in the inferior leads with corresponding positive flutter wave in lead V1, with a regular atrial rate between 240 and 340 bpm on 12-lead ECG or 24-h Holter (Fuster et al., 2011). Atrial fibrillation was diagnosed when ECG shows the replacement of regular p waves with uncoordinated fibrillatory waves with an irregular ventricular rate and lasting for 1 min at least (Fuster et al., 2011). The CHA_2_DS_2_-VASc score [congestive heart failure (1 point), hypertension (1 point), age ≥65 (1 point), age ≥75 (2 points), diabetes mellitus (1 point), prior stroke or transient ischemic attack (TIA) (2 points), vascular disease (1 point), female (1 point)] and HATCH score [hypertension (1 point), age ≥75 (1 point), prior stroke or TIA (2 points), chronic obstructive pulmonary disease (1 point), and heart failure (1 point)] were calculated for each patient.

### Electrophysiology Study and Catheter Ablation

All AADs except amiodarone were discontinued for at least five half-lives before the procedure, and low-molecular-weight heparin was administered subcutaneously 3–5 days until the procedure day. The transesophageal echocardiogram was performed to rule out left atrial (LA) thrombus in each patient. The radiofrequency catheter ablation was used to perform a linear lesion between the tricuspid annulus and inferior vena cava for bidirectional conduction block at CTI. The tachycardia was confirmed with the CARTO system (Biosense Webster, Diamond Bar, CA, United States) and 3.5-mm tip ablation catheter (NAVISTAR THERMOCOOL, Biosense Webster) with a target temperature of 43°C and power of 35 W, and infusion rate of 17 ml/min was applied for CTI ablation. The successful ablation was defined by CTI block lasting for at least 20 min after the last radiofrequency application.

### Follow-Up

All patients underwent continuous ECG monitoring for at least 24 h after CTI ablation. No AADs were prescribed postablation. The warfarin was stopped at 3 months in patients with CHA_2_DS_2_-VASc = 2 if no arrhythmia recurrence and continued in CHA_2_DS_2_-VASc ≥ 2 throughout the study period after ablation. Each patient was routinely followed up at 1, 3, 6, and 12 months (after index ablation procedure) and every 6 months until arrhythmia recurrence including AFL and AF by 12-lead ECG and 24-h Holter. Additionally, 12-lead ECG was advised if any patient became symptomatic after the index ablation procedure. The clinical endpoint was new-onset AF.

### Statistical Analysis

Statistical analysis was performed by SPSS 24.0 (IBM, Armonk, NY, United States). The Student *t*-test was used for continuous variable and presented as mean ± SD. For categorical variables, χ^2^ or Fisher exact test was used for comparison analysis and presented as a proportion. Cox proportional models were analyzed for predictors of postablation new-onset AF. Statistically significant factors in the univariate analysis were selected for multivariate analysis. Kaplan–Meier analysis with a log-rank test was performed to determine the difference of CHA_2_DS_2_-VASc score as related to the cumulative risk of new-onset AF. Additionally, a receiver operating characteristic (ROC) curve was constructed to test the ability of the CHA_2_DS_2_-VASc and HATCH scores to predict new-onset AF.

## Results

### Clinical Characteristics

Bidirectional CTI block was successfully achieved in all patients. Of 124 cases, 13 patients either refused or did not attend follow-up and eight patients experienced AFL recurrence, thus 103 patients were included in the final analysis. The baseline characteristics of the participants are shown in Table 1. The mean age of the patients was 60 ± 16 years. Majority of the patients were males, 82 (79.6%). The most prevalent comorbidity was hypertension (25.2%), followed by diabetes mellitus (18.4%), cardiac failure (16.5%), and ischemic heart disease (9.7%). The mean LA diameter was 36.6 ± 6.8 mm, and the mean left ventricular ejection fraction (LVEF) was 60.4 ± 11.4%. The average CHA_2_DS_2_-VASc score was 1.73 ± 1.4 points, and score distribution for 0, 1, 2, and ≥3 points were 22.3, 25.2, 25.2, and 27.3%, respectively. The group with higher new-onset AF tended to be older, carry the burden of hypertension, prior stroke/TIA, and enlarged LA dimension as compared to without AF patients (*P* < 0.05; Table 1).

**TABLE 1:** Baseline characteristics.

**Variables**	**All (103)**	**AF (*n* = 33)**	**No AF (*n* = 70)**	***P*-value**
Age (years ± SD)	60.3 ± 16.0	68.7 ± 12.4	56.4 ± 16.1	**<0.001**
Male	82(79.6%)	24(72.7%)	58(82.9%)	0.234
Medical history				
Hypertension	26(25.2%)	13(39.4%)	13(18.6%)	**0.023**
Diabetes mellitus	19(18.4%)	7(21.2%)	12(17.1%)	0.619
Ischemic heart disease	10(9.7%)	3(9.1%)	7(10%)	0.884
Heart failure	17(16.5%)	6(18.2%)	11(15.7%)	0.753
Previous stroke/TIA	8(7.8%)	7(21.2%)	1(1.4%)	**0.001**
Vascular disease	8(7.8%)	4(12.1%)	4(5.7%)	0.257
COPD	8(7.8%)	5(15.2%)	3(4.3%)	0.126
Echocardiogram characteristics				
LAD, mm	36.6 ± 6.8	39.3 ± 5.4	35.3 ± 7.1	**0.006**
LVEF, %	60.4 ± 11.4	59.3 ± 11.9	60.9 ± 11.2	0.531
AAD use before ablation	42(40.8%)	16(48.5%)	26(37.1%)	0.274
Beta-blocker	10(9.7%)	3(9.1%)	7(10%)	0.884
Calcium channel blocker	6(5.8%)	4(12.1%)	2(2.9%)	0.073
Propafenone	14(13.6%)	7(21.2%)	7(10%)	0.132
Amiodarone	12(11.7%)	3(9.1%)	11(15.7%)	0.345
ACEI/ARB	10(9.7%)	4(12.1%)	6(8.6%)	0.577
Digitalis	8(7.8%)	3(9.1%)	5(7.1%)	0.749
CHA_2_DS_2_-VASc score	1.73 ± 1.40	2.79 ± 1.45	1.23 ± 1.07	**<0.001**
HATCH score	1.06 ± 1.17	1.82 ± 1.45	0.70 ± 0.81	**<0.001**

*Data are shown as mean ± standard deviation for continuous variables and as number (%) for categorical variables. The *P*-values with a bold font are significant unless otherwise stated. AF, atrial fibrillation; TIA, transient ischemic attack; COPD, chronic obstructive pulmonary disease; LAD, left atrial diameter; LVEF, left ventricular ejection fraction; AAD, anti-arrhythmic drug; ACEI, angiotensin-converting enzyme inhibitor; ARB, angiotensin II receptor blocker.*

### Occurrence and Predictors of New-Onset Atrial Fibrillation After Atrial Flutter Ablation

After 24.6 ± 16.9 months’ follow-up period, 33 (32%) patients experienced an episode of AF. Among them, 10 patients (30.3%) experienced AF episodes within the first 6 months and the remaining 23 (69.7%) after 6 months of the ablation procedure. The median duration for the occurrence of a new episode of AF was 7 months after CTI ablation. Multivariate Cox regression model showed that the CHA_2_DS_2_-VASc score [hazard ratio (HR) = 1.736, 95% confidence interval (CI) = 1.370–2.201, *P* < 0.001] and HATCH score (HR = 1.459, 95% CI = 1.136–1.873, *P* = 0.003) were independently associated with the incidence of new-onset AF (Table 2).

**TABLE 2:** Cox regression analysis for predictors of new-onset atrial fibrillation after atrial flutter ablation.

**Variables**	**Univariate analysis**			**Multivariate analysis**		

	**HR**	**95% CI**	***P*-value**	**HR**	**95% CI**	***P*-value**
Age (years)	1.049	1.020-1.080	**0.001**			
Gender	1.651	0.767-3.555	0.200			
Heart failure	1.063	0.439-2.576	0.892			
Hypertension	2.144	1.064-4.320	**0.033**			
Diabetes mellitus	1.200	0.521-2.765	0.669			
Previous stroke/TIA	4.836	2.080-11.242	**<0.001**			
Vascular disease	1.864	0.643-5.298	0.254			
COPD	2.973	1.141-7.747	**0.026**			
LAD (mm)	1.068	1.023-1.114	**0.002**			
CHA_2_DS_2_-VASc score	1.775	1.414-2.227	**<0.001**	1.736	1.370-2.201	<0.001*
HATCH Score	1.655	1.314-2.085	**<0.001**	1.459	1.136-1.873	0.003*

*Adjusted for age, hypertension, previous stroke/TIA, COPD, and LAD. The *P*-values with a bold font are significant unless otherwise stated. CI, confidence interval; HR, hazard ratio; TIA, transient ischemic attack; COPD, chronic obstructive pulmonary disease; LAD, left atrial diameter.

### CHA_2_DS_2_-VASc Score for Prediction of New-Onset Atrial Fibrillation After Atrial Flutter Ablation

**FIGURE 1:**
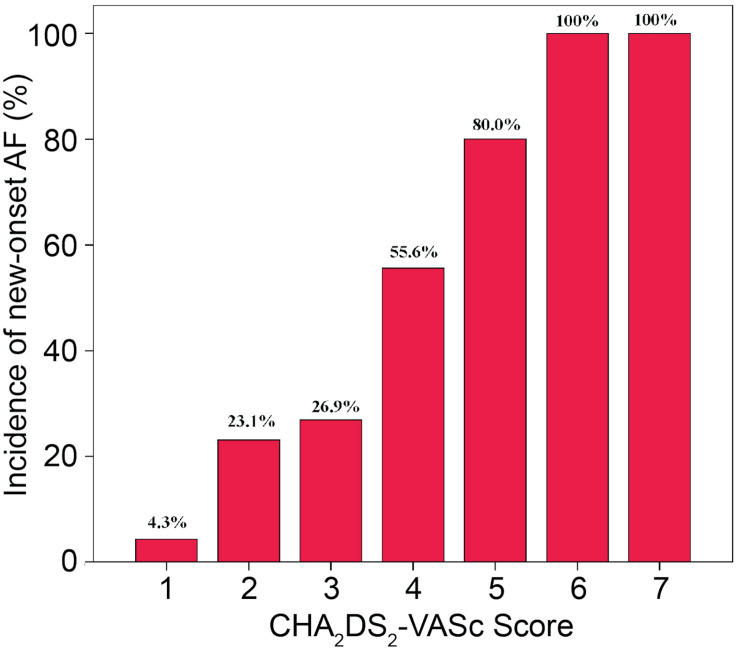
Incidence of new-onset atrial fibrillation and CHA_2_DS_2_-VASc score.

**FIGURE 2:**
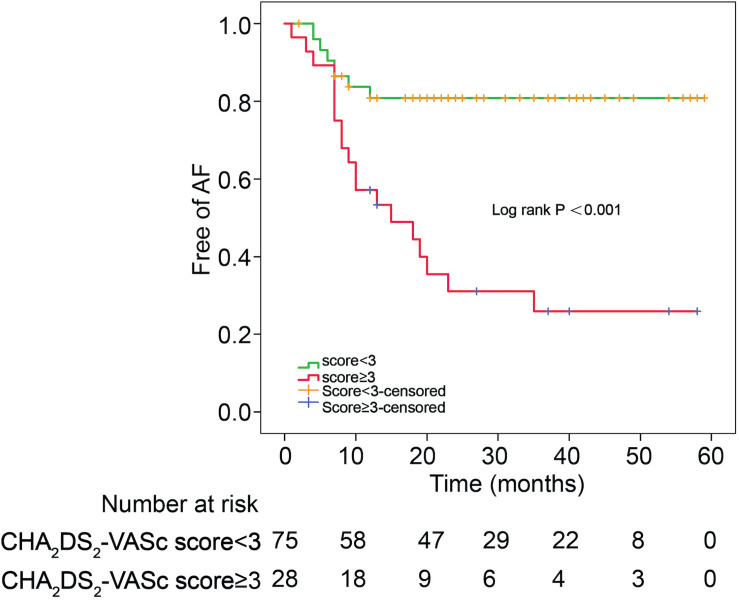
Atrial fibrillation-free survival curves for patients with different CHA_2_DS_2_-VASc scores.

**FIGURE 3:**
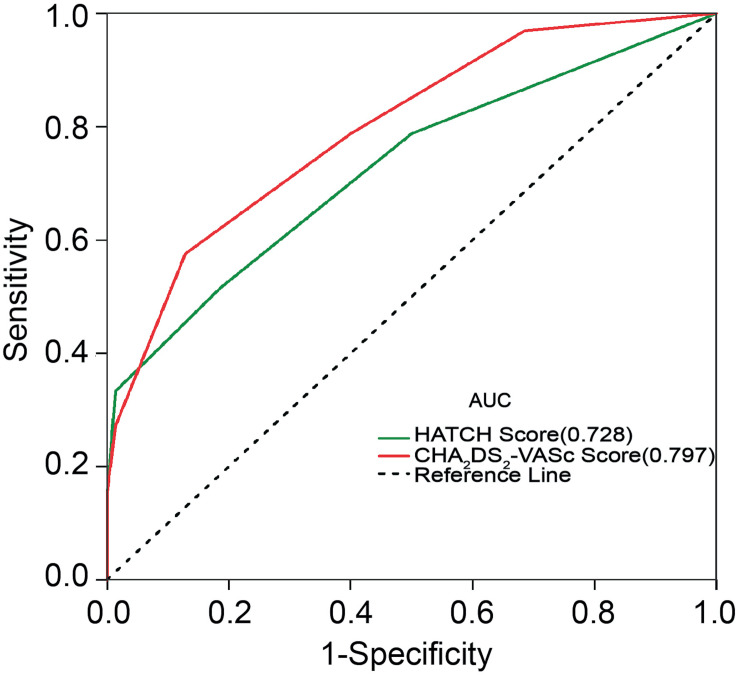
Receiver operating characteristic curve for prediction of atrial fibrillation with the CHA_2_DS_2_-VASc score and HATCH score. AUC, area under the curve.

The incidences of AF in patients with CHA_2_DS_2_-VASc score ≥3 and CHA_2_DS_2_-VASc score <3 were 67.9 and 18.7%, respectively (*P* = 0.001). Table 3 illustrates the baseline characteristics between patients with CHA_2_DS_2_-VASc scores <3 and ≥3. Patients with higher CHA_2_DS_2_-VASc score developed new-onset AF more often and associated with shorter duration as shown in Figures 1, 2 (Kaplan–Meier survival analysis). The CHA_2_DS_2_-VASc scores predicted the new-onset AF with the ROC curves displaying sensitivity and specificity of 57.6 and 87.1%, respectively. The sensitivity and specificity of HATCH score at the cutoff point of 2 were 51.5 and 81.4%, respectively (Figure 3). Also, the prediction analyses of new-onset AF episodes based on CHA_2_DS_2_-VASc scores at a cutoff point of 3 and HATCH score at the cutoff point of 2 yielded an area under the curve (AUC) of 0.797 and 0.728, respectively.

**TABLE 3:** Baseline characteristics in patients with different CHA_2_DS_2_-VASc scores.

**Variables**	**CHA_2_DS_2_-VASc score ≥ 3 (*n* = 28)**	**CHA_2_DS_2_-VASc score < 3 (*n* = 75)**	***P*-value**
AF	19(67.9%)	14(18.7%)	<0.001
Age (years)	75 ± 5.6	54 ± 15.1	<0.001
Male	22(78.6%)	60(80%)	0.873
Medical history			
Hypertension	12(42.9%)	14(18.7%)	0.012
Diabetes mellitus	12(42.9%)	7(9.3%)	<0.001
Ischemic heart disease	5(17.9%)	5(6.7%)	0.105
Heart failure	4(23.5%)	13(12.4%)	0.711
Previous stroke/TIA	8(28.6%)	0(0.0%)	<0.001
Vascular disease	5(17.9%)	3(4.0%)	0.029
COPD	3(10.7%)	5(6.7%)	0.788
Echocardiogram characteristics			
LAD, mm	37.1 ± 4.9	36.3 ± 7.4	0.632
LAD, ≥35 mm	20(80%)	40(56.3%)	0.036
LVEF, %	60.9 ± 10.3	60.2 ± 11.9	0.803
AAD use before ablation	9(32.1%)	33(44.0%)	0.276
Beta-blocker	2(7.1%)	8(10.7%)	0.870
Calcium channel blocker	4(14.3%)	2(2.7%)	0.077
Amiodarone	6(21.4%)	8(10.7%)	0.172
Propafenone	3(10.7%)	11(14.7%)	0.595
ACEI/ARB	5(17.9%)	5(6.7%)	0.105
Digitalis diuretic agents	1(3.6%)	7(9.5%)	0.289

*AF, atrial fibrillation; TIA, transient ischemic attack; COPD, chronic obstructive pulmonary disease; LAD, left atrial diameter; LVEF, left ventricular ejection fraction; AAD, anti-arrhythmic drug; ACEI, angiotensin-converting enzyme inhibitor; ARB, angiotensin II receptor blocker.*

## Discussion

To the best of our knowledge, this is the first study to evaluate the ability and utilization of CHA_2_DS_2_-VASc score as a predictor of a new-onset AF after CTI ablation of typical AFL. The present study demonstrated that those patients with CHA_2_DS_2_-VASc score ≥3 had a higher likelihood of developing new-onset AF post-CTI ablation. The CHA_2_DS_2_-VASc score ≥3 was associated with a significant risk for new-onset AF post-CTI ablation, with slightly better predictive power compared with HATCH score ≥2.

According to previously published data, a significant proportion of patients undergoing CTI ablation for AFL develop new-onset AF during follow-up (Enriquez et al., 2015; Celikyurt et al., 2017). The present study detected that nearly one-third of the patients developed new-onset AF during 24.6 ± 16.9 months of follow-up after CTI ablation, consistent with the previously reported incidence (25%) (Celikyurt et al., 2017). Previous studies have identified several risk factors for developing AF after typical AFL ablation, including LA enlargement, a history of AF, reduced LVEF and AF inducibility (Brembilla-Perrot et al., 2014; Joza et al., 2014; Voight et al., 2014), while other variables such as age, hypertension, LA size, LV systolic dysfunction, a history of AF, or structural heart disease did not reach statistical significance for prediction (Chinitz et al., 2007). These conflicting reports may be due to the small number of patients in these studies.

Earlier evidence reported that HATCH score is an effective predictive model to estimate the risk of postablation AF in AFL patients (Chen et al., 2015). Similarly, the present study confirmed that CHA_2_DS_2_-VASc and HATCH scores are associated with postablation new-onset AF in typical AFL patients. However, our findings show that the CHA_2_DS_2_-VASc score (AUC of 0.797) had a slightly stronger predictive power compared with the HATCH score (AUC of 0.728) in our study. This could be attributed to the fact that the HATCH model does not include additional risk factors such as gender, diabetes, and vascular disease. These additional components of CHA_2_DS_2_-VASc score have been reported as independent risk factors for the development of AF (Lau et al., 2017). A number of studies demonstrated the CHA_2_DS_2_-VASc score as a significant predictor for adverse events and as a risk factor of AF recurrence in patients undergoing AF catheter ablation (Kornej et al., 2014; Wang et al., 2016). Moreover, Park et al. (2011) proved that the electroanatomical remodeling estimated by LA volume and endocardial voltage had a significant relationship with CHA_2_DS_2_-VASc score in AF patients. Also, Ribo et al. (2015) concluded that a high CHA_2_DS_2_-VASc score promotes extensive AF substrate. These evidences suggest that the high CHA_2_DS_2_-VASc score may associate with the electroanatomical remodeling of the atrium in AF patients.

Recently, Romero et al. (2017) showed that AF inducibility by atrial burst pacing and extra-stimulation post-CTI ablation was highly predictive of subsequent AF occurrence. Similarly, we identified the CHA_2_DS_2_-VASc score ≥3 as an independent and strong predictor of new-onset AF after CTI ablation in typical AFL patients. In contrast, AAD therapy before AFL ablation was irrelevant to new-onset AF, which is inconsistent with the previous observation (Brembilla-Perrot et al., 2014). In our study, patients with a CHA_2_DS_2_-VASc score ≥3 had an advanced age, a greater history of hypertension, diabetes mellitus, and vascular heart disease. Also, patients with CHA_2_DS_2_-VASc score ≥3 had a history of previous stroke/TIA and larger left atrium size. This may further suggest that AFL patients with CHA_2_DS_2_-VASc score ≥3 undergo substantial electroanatomical alterations. Also, an earlier study suggested the use of atrial electrograms to help identify patients with atrial myopathy, a condition that may exist without AF and can facilitate the development of AF (Shen et al., 2019). The findings from our study along with the previous studies suggest that a combination of extensive electrophysiological evaluation of atrial electrograms along with AF inducibility by atrial burst pacing in AFL patients with CHA_2_DS_2_-VASc score ≥3 may efficiently identify patients at highest risk for AF.

There is a close pathophysiologic relationship between AFL and AF (Waldo and Feld, 2008). Pulmonary vein triggers have been proposed to play an essential role in patients with AFL, and prophylactic PVI can reduce new-onset AF in patients with isolated AFL (Joza et al., 2014; Mohanty et al., 2015; Schneider et al., 2015; Romanov et al., 2018). However, the risk/benefit ratio of prophylactic PVI is still controversial. Gula et al. (2016) conducted a cost–benefit analysis for lone AFL, comparing the strategy of combined CTI + PVI to that of sequential procedures. The combined approach with prophylactic PVI conferred greater risk and higher cost than the sequential approach. Perhaps a strategy of combined CTI + PVI would have a more reasonable benefit/risk ratio if applied particularly in patients at the highest risk for AF. Considering CHA_2_DS_2_-VASc score as a strong predictor of new-onset AF post-CTI ablation particularly ≥3 scores, conducting a cost–benefit analysis for lone AFL with CHA_2_DS_2_-VASc scores ≥3 would be meaningful. Previously, the observational study confirmed that patients with AFL had a higher rate of all-cause mortality and similar thromboembolic events compared with those with AF after PVI (Vadmann et al., 2017). Therefore, there is an urgent need for an efficient system to predict the occurrence of postablation AF in AFL patients particularly to guide future management.

## Limitations

There are several limitations to the present study. Firstly, the retrospective nature with a small sample size that was carried out in a single center. Secondly, no implantable loop monitoring was performed before and after CTI ablation, which may lead to underestimation of prior and postablation AF occurrence. Therefore, large prospective studies with continuous ECG monitoring prior to ablation and postablation in AFL patients are required to confirm our findings.

## Conclusion

The CHA_2_DS_2_-VASc score is a useful tool for the prediction of new-onset AF after ablation of typical AFL. Patients with CHA_2_DS_2_-VASc score ≥3 are more likely to develop new-onset AF and should be monitored more closely.

## Data Availability Statement

The datasets generated for this study are available on request to the corresponding author.

## Ethics Statement

The studies involving human participants were reviewed and approved by The First Affiliated Hospital of Dalian Medical University Ethics committee. Written informed consent for participation was not required for this study in accordance with the national legislation and the institutional requirements.

## Author Contributions

XY and YX designed the study. FL, ZX, and KB contributed in the study protocol, literature searches, data collection, and statistical analysis and were involved in the final draft of the manuscript. YL, AL, YS, GT, and LG contributed in the coordination of designing and analysis. All authors have read and approved the final manuscript.

## Conflict of Interest

The authors declare that the research was conducted in the absence of any commercial or financial relationships that could be construed as a potential conflict of interest.
